# Molecular Characterization of Oral Squamous Cell Carcinoma in Mexican Patients: A Genomic and Epidemiological Overview

**DOI:** 10.3390/cancers17203282

**Published:** 2025-10-10

**Authors:** Javier Lopez-Gomez, Dennis Cerrato-Izaguirre, Ericka Marel Quezada-Maldonado, L. A. Gaitan-Cepeda, Mauricio Salcedo, Marco Antonio Hernandez-Castillo, Martín Granados-García, Yesennia Sánchez-Pérez, Claudia M. García-Cuellar

**Affiliations:** 1 Dirección de Investigación, Instituto Nacional de Cancerología, San Fernando No. 22, Tlalpan, Ciudad de México CP 14080, Mexico; dr.javierlopgom@gmail.com; 2 Dirección Médica, Hospital IMSS Bienestar, Cuajimalpa CP 05500, Mexico; 3 Departamento de Cirugía Oncológica, Hospital de Oncología de Centro Médico Nacional s.XXI (Instituto Mexicano del Seguro Social, IMSS), Ciudad de México CP 06720, Mexico; 4 Subdirección de Investigación Básica, Instituto Nacional de Cancerología, San Fernando No. 22, Tlalpan, Ciudad de México CP 14080, Mexico; dennis_cerrato@yahoo.com (D.C.-I.); marelquezada0612@gmail.com (E.M.Q.-M.); ysanchezp@incan.edu.mx (Y.S.-P.); 5 Departamento de Patología Oral y Medicina Oral, División de Posgrado e Investigación, Facultad de Odontología, Universidad Nacional Autónoma de México, Ciudad de México CP 04510, Mexico; lgaitan@unam.mx; 6 Unidad de Investigación en Biomedicina y Oncología Genómica, Hospital de Gineco-Pediatría 3A, OOAD CDMX Norte, Instituto Mexicano del Seguro Social (IMSS), Ciudad de México CP 08230, Mexico; masava89@gmail.com; 7 Departamento de Estomatología, Complejo Regional Sur, Benemérita Universidad Autónoma de Puebla, Puebla CP 72000, Mexico; markhercas12@gmail.com; 8 Departamento de Cabeza y Cuello, Instituto Nacional de Cancerología, San Fernando No. 22, Tlalpan, Ciudad de México CP 14080, Mexico; martingranadosmx@gmail.com

**Keywords:** oral squamous cell carcinoma, prognosis, biomarkers, tumorigenesis, mutation

## Abstract

Background/Objectives: Oral squamous cell carcinoma (OSCC) is a major global malignancy caused by tobacco and alcohol consumption, human papillomavirus (HPV) infection, and genetic alterations. Emerging genomic insights have revealed recurrent mutations that influence tumorigenesis, prognosis, and the effectiveness of targeted therapies. Although prevalent, these mutations have not yet been described in the Mexican population. This study explored the molecular landscape of OSCC in Mexican patients, addressing the gaps in genomic research and postulating potential clinical implications. Methods: A prospective cohort study of 55 patients with OSCC was conducted (2018 to 2025). Whole-exome sequencing identified genomic alterations, tumor mutational burden, mutational signatures, disrupted signaling pathways, and the presence of HPV and Epstein–Barr Virus (EBV). Correlation of molecular alterations with prognostic and therapeutic indicators were performed. Results: *TP53* (51%, *p* = <0.001), *FAT1* (20%, *p* = 0.027), and *KMT2C* (18%, *p* = 0.003) were identified as potential predictors of response to immunotherapy. SBS 5 (*p* = 0.016) and ID-19 (*p* < 0.001) signatures, characteristic of the Mexican population, were correlated with prognosis and periodontal disease (*p* = 0.001). A genetically distinct OSCC subgroup (31%), primarily young women with advanced disease, lacked traditional risk factors and exhibited a high ID-19 prevalence (*p* = 0.011). *CYTH4* mutations and NOTCH pathway activation were uniquely identified (*p* = 0.026). *TP53*, *FAT1*, *KMT2C*, SBS 5, and ID-19 were identified as potential biomarkers for risk stratification and therapy selection. The non-smoking, non-drinking subgroup exhibited NOTCH pathway activation, *CYTH4* mutation, and ID-19 prevalence, suggesting alternative oncogenic mechanisms. Conclusions: These findings support molecular-driven OSCC classification and emphasize precision medicine for personalized treatment and prognosis improvement.

## Simple Summary

Oral cancer often develops due to smoking, alcohol use, or viral infections. However, many patients in Mexico are diagnosed at an advanced stage, and some do not have these traditional risk factors, suggesting that other causes may be involved. In this study, we analyzed the genetic makeup of tumors obtained from Mexican patients with oral cancer to better understand the disease. We identified significant changes in specific genes and discovered unique genetic patterns associated with inflammation and periodontal diseases. We also identified a distinct group of patients, mostly younger women without smoking or drinking habits, whose cancers follow different biological pathways. These findings suggest that oral cancer in Mexico may have unique features compared with other populations. Understanding these differences can help doctors improve early detection, predict outcomes more accurately, and provide personalized treatments for patients.

## 1. Introduction

Oral squamous cell carcinoma (OSCC) is one of the most common malignancies affecting the head and neck and account for over 90% of all oral cancers (1). Globally, 377,713 new OSCC cases and 177,757 OSCC-related deaths were reported in 2020 (2). In Mexico, OSCC contributes to 1500 new cases and 586 deaths annually, positioning it 21st in terms of incidence and 24th in terms of mortality. However, these figures may be underestimated because of incomplete cancer registries in some regions [1,2]. This malignancy primarily affects men over 50 years of age, with over 90% of cases being OSCCs [3]. OSCC is strongly associated with tobacco and alcohol consumption, Human papillomavirus (HPV) infection, chronic inflammation, areca (betel) nut chewing, and genetic predisposition [4,5,6,7,8,9,10,11]. OSCC typically presents as painful, rapidly growing ulcers in the oral cavity. Advanced cases may present with trismus, tongue immobility, or cervical lymphadenopathy. Diagnosis relies on physical examination, histopathological biopsy, and imaging studies [3,12,13]. Early-stage OSCC accounts for 29% of cases in the U.S.; however, 70% of cases in Mexico are diagnosed at advanced stages [12,13,14]. The management of OSCC requires a multidisciplinary approach, with surgical resection as the primary therapeutic modality. Adjuvant radiotherapy or chemoradiotherapy is commonly employed in locally advanced diseases. Metastatic OSCC is managed with systemic therapy, including chemotherapy, targeted therapy, or immunotherapy [3]. Advances in next-generation sequencing (NGS) have identified recurrent genomic alterations that contribute to OSCC tumorigenesis, presenting potential biomarkers for early detection and targeted therapy [15,16].

Chronic inflammation of the oral mucosa is recognized as a promoter of OSCC. Persistent inflammatory stimuli, such as periodontal disease, poor oral hygiene, or prolonged infection, generate a microenvironment rich in reactive oxygen and nitrogen species, proinflammatory cytokines, and growth factors. These mediators induce oxidative DNA damage, activate transcription factors, such as *NF-κB* and *STAT3*, and drive epigenetic modifications that together promote genomic instability, uncontrolled proliferation, and resistance to apoptosis. Repeated tissue injury and repair further enhance angiogenesis and epithelial–mesenchymal transition, creating conditions conducive to malignant transformation. Clinical studies have linked chronic periodontitis and other inflammatory oral disorders with an increased risk of OSCC, supporting the concept that sustained inflammation acts as both an initiator and promoter of carcinogenesis [17,18]

Oral carcinogenesis involves genetic and epigenetic alterations that disrupt oncogenes and tumor suppressor genes, leading to uncontrolled cell proliferation and tumor invasiveness. Whole-exome sequencing (WES) has facilitated the identification of key driver mutations that can serve as potential biomarkers for risk stratification, early detection, and targeted therapy development [15,16]. A TCGA cohort of 311 OSCC samples identified recurrent mutations in *TP53*, *FAT1*, *NOTCH1*, *CASP8,* and *PIK3CA*. However, Latino populations are underrepresented in genomic studies [11,19,20,21]. Distinct mutational patterns are observed across ethnicities: Indian populations carry mutations in *USP9X*, *KMT2B*, *ARID2*, *UNC13C*, and *TRPM3*, whereas Arab populations exhibit frequent alterations in *CLTCL1*, *TRPM,* and *OSMR*, with a 70% prevalence of Epstein–Barr Virus (EBV) [11,19,20,21]. In contrast, mutations in *FAT1* predominate in Asian populations, whereas in Taiwan, *CASP8* and *FAT1* mutations occur frequently, with a lower incidence of *TP53* mutations, suggesting distinct molecular drivers in this region [16]. Recent studies in Australia, India, Taiwan, and Pakistan have also identified significant genetic variability, highlighting mutations in a wide range of genes [22,23]. The U.S. Oral Tongue Cancer Consortium analyzed 227 samples, mostly from a Caucasian population, and detected low tumor mutational burden (TMB) in patients under 50 years of age [24] (Table S1).

Mutation signature analysis provides insight into DNA damage and repair mechanisms. Alexandrov et al. identified 72 single-base substitution (SBS) signatures and 18 insertion–deletion (Indel) signatures [25,26,27,28,29]. Key SBS signatures in OSCC include SBS1, SBS4, SBS5, SBS2/SBS13, ID1, and ID2/ID3 [28,29].

The presence of lymphatic metastases, depth of invasion (DOI), tumor stage, and surgical margin status were the most important prognostic factors. Genomic studies have identified *FAT1*, *NOTCH1*, and *CASP8* as prognostic markers [19]. *CDKN2A* mutations are associated with poor OS. Co-occurring alterations in *TP53*, *NOTCH*, and *RTK/RAS/MAPK* pathways significantly affected survival outcomes. Notably, combinations such as the Hippo–NOTCH and NOTCH–PI3K signaling pathways correlate with improved disease-free survival [25]. The 5-year overall survival rates are 80–85% for early stage OSCC, 50–65% for locally advanced OSCC, and 20–30% for metastatic OSCC [30,31]. This study aimed to determine the mutational profile, identify the most affected signaling pathways, detect the presence of HPV and EBV, characterize the mutational signatures and TMB, and correlate the molecular alterations with the clinical and pathological parameters of Mexican patients with OSCC.

Significance: *TP53*, *FAT1*, *KMT2C*, SBS 5, and ID-19 are key prognostic biomarkers, along with a genetically distinct subgroup of non-smoking and non-drinking patients. The findings highlight alternative oncogenic pathways and periodontal disease associations, and support molecular-driven classification to improve prognosis, immunotherapy response, and personalized treatment strategies.

## 2. Materials and Methods

### 2.1. Dataset

Since OSCC has a relatively low incidence in Mexico (21st in incidence and 24th in mortality) [1,2], the Instituto Nacional de Cancerología (INCan) sees only a limited number of new cases each year. Accordingly, we designed a prospective pilot cohort study to characterize genomic alterations in OSCC by, consecutively recruiting Mexican patients between December 2018 and February 2025 who met the following criteria: age ≥ 18 years, histologically confirmed squamous cell carcinoma, no prior oncologic treatment, and provision of written informed consent. Exclusion criteria included insufficient tumor or adjacent tissue samples (<0.5 cm), loss to follow-up, uncertain primary tumor origin (oropharyngeal vs. oral cavity), and a DNA Integrity Number (DIN) < 6. A comprehensive clinical evaluation was conducted for all participants, including the collection of demographics (age, sex) and clinical–pathological data such as tumor subsite, disease staging (AJCC 8 edition), DOI, and survival. Additionally, lifestyle factors, such as tobacco and alcohol consumption, use of dental prostheses, and periodontal disease were recorded. Tumor and adjacent healthy tissue biopsies were obtained. All biological samples were snap-frozen in liquid nitrogen, stored at −80 °C, and then transported to the research laboratory at the INCan for DNA extraction and WES. The study protocol was reviewed and approved by the INCan Research and Ethics Committee (Approval Numbers: 018/086/IBI and CEI/1316/18).

### 2.2. DNA Extraction and Whole-Exome Sequencing

DNA was extracted from tumors and adjacent tissues and sequenced at Novogene Inc. (Sacramento, CA, USA) using the SureSelect Human All Exon V6 Kit (Agilent, 5190-8865, Santa Clara, CA, USA) following their standardized methodology. High-integrity (≥1 µg) DNA was fragmented into 150–200 base pair (bp) segments, polyadenylated at the 3′ end, and ligated to polymerase-compatible adapters. Exonic regions were captured using biotin-labeled probes and streptavidin-coated magnetic beads, followed by purification using the AMPure XP system (Beckman Coulter, A63882, Indianapolis, IN, USA). The libraries were amplified, hybridized, and quantified using an Agilent Bioanalyzer 2100 (Agilent technologies, Santa Clara, CA, USA). Sequencing was performed using the Illumina NovaSeq 6000 system (Ilumina, Inc. San Diego, CA, USA) with sequencing-by-synthesis technology, generating FASTA-format files for downstream bioinformatic analysis.

### 2.3. Somatic Mutation Calling

Raw sequencing data were quality filtered to remove adapter sequences and low-quality reads. The reads were discarded if they contained adapter contamination, >10% uncertain bases, or >50% low-quality bases. Reads were aligned to the human reference genome (GRCh37/hg19) using the Burrows–Wheeler Aligner (BWA-MEM v0.7.8-r455) [32]. BAM files were generated using SAMtools (v1.0), and duplicate reads were removed using Picard (v1.111). Picard, part of the Genomic Analysis Toolkit (GATK, Broad Institute, Cambridge, MA, USA), enhances variant calling accuracy by correcting misalignments [33,34]. Single-nucleotide variants (SNVs) were identified using MuTect (v1.1.4) [35], VarScan2 (v4.1) [36], and MuTect2 (v4.2) [37]. Only the SNVs identified by at least two variant callers were considered. Insertions and deletions (InDels) were identified using Strelka (v1.0.13), VarScan2, and MuTect2. InDels were retained only if they were detected by at least two independent variant callers. Variants with a minor allele frequency (MAF) ≥ 1% (gnomAD v2.0.1) [38], variant allele fraction (VAF) < 0.05 or >0.7, and shared mutations between tumor and normal tissue (paired-sample analysis) were excluded.

### 2.4. Copy-Number Alteration Analysis

Control-FREEC (v11.4) was used to identify copy number variations (CNVs) in chromosomal cytobands. This tool, developed by the Institute Curie, differentiates between somatic and germline CNV events. CNV classification was based on log2 ratios: CNV gains/Log2 ratio > 0.2 and CNV losses/Log2 ratio < −0.235 [39].

### 2.5. Tumor Mutational Burden (TMB) and Driver Mutation Identification

Germline variants from dbSNPs were excluded. TMB classification (high vs. low) was determined using CutoffFinder V1 [40]. Driver Mutations were identified using the Cancer Genome Interpreter (CGI), applying a tissue-specific model (gastric adenocarcinoma) with the BoostDM machine learning algorithm. Mutations with a BoostDM score ≥ 0.5 and an F50 predictive score > 0.9 were classified as oncogenic drivers [41].

### 2.6. Pathway Enrichment Analysis

Functional pathway enrichment analysis was performed using WebGestalt v.2024 (a WEB-based Gene Set Analysis Toolkit, Columbus, OH, USA). Overrepresentation analysis identified significantly altered signaling pathways based on the Reactome and Panther Pathway databases. Pathways with a false discovery rate (FDR) < 0.05 were considered statistically enriched [42,43,44].

### 2.7. Mutational Signature Analysis

Mutational signature analysis was performed using both version 2 (V2) and version 3 (V3). Known germline variants reported in dbSNP were excluded from both analyses. V2 signatures were analyzed using R (v3.5.1 Feather Spray) and the deconstructSigs package [45], and V3 signatures were analyzed using the “SigProfiler Bioinformatics Tool” from COSMIC and subsequently grouped based on unsupervised clustering of mutational signature similarities [46].

### 2.8. HPV and EBV Detection via qRT-PCR

HPV and EBV detection were performed using quantitative real-time polymerase chain reaction (qRT-PCR) [47]. DNA (100 ng) was amplified using RealQ Plus 2x Master Mix Green, High ROX (Ampliqon, Odense, Denmark). Universal Primers Gp5/Gp6 were used to screen for multiple HPV types [6,16,20,22]. HPV16 and HPV18-specific primers were subsequently used for further characterization of the positive samples. A single primer set was used to detect EBV. The SIHA (HPV16) and HELA (HPV18) cell lines and confirmed EBV-positive gastric cancer samples were used as positive controls for HPV and EBV, respectively. β-Globin gene amplification was performed as an internal quality check.

### 2.9. Statistical Analysis

Descriptive statistics were used to summarize the data. Continuous variables were described using measures of central tendency (mean or median) and dispersion (standard deviation or interquartile range), depending on their distribution and categorical variables were expressed as absolute frequencies and percentages. Associations between frequently mutated genes and clinical characteristics, with particular attention paid to alcohol and tobacco consumption, were evaluated using a combination of bivariate and multivariate methods. Categorical variables (e.g., mutation status and lymph-node metastases) were compared using Chi-square or Fisher’s exact tests (when the expected frequencies were <5). Parametric continuous variables (e.g., TMB and tobacco index) were analyzed using Student’s *t*-test, and non-parametric variables (e.g., depth of invasion) were analyzed using the Mann–Whitney *U* test. Ordinal variables were treated as categorical variables and assessed using the Chi-square test. Overall survival (OS) and disease-free survival (DFS) analyses were conducted using the Kaplan–Meier method, with log-rank tests to compare curves. To identify independent prognostic factors, Cox proportional hazards regression models were fitted, incorporating genomic features such as TMB, mutational signatures, and clinically relevant genes. Variables included in the Cox models were selected based on the prognostic relevance reported in the literature or identified in univariate analyses. The results are presented as hazard ratios (HRs) with 95% confidence intervals (CIs). A two-sided significance level of 0.05 was applied to all tests. All statistical analyses were performed using SPSS v27 (2021).

## 3. Results

### 3.1. Demographic Data

A total of 55 patients diagnosed with OSCC between December 2018 and February 2025, with a median follow-up of 27.9 months (range: 1.6–73.4 months), were included in this study. The cohort comprised 30 males and 25 females with a mean age of 61.3 years (range: 21–86 years). The detailed demographic and clinicopathological characteristics are summarized in Table 1 and Table 2, respectively.

### 3.2. Tumor Staging

The tumor subsite distribution according to the AJCC classification is detailed in Table 2; the mobile tongue was the highest contributor (45.5%). Regarding clinical staging, patients were classified as follows: Stage 0 (carcinoma in situ): one patient (1.8%); Stage I: three patients (5.5%); Stage II: five patients (9.1%); Stage III: five patients (9.1%); Stage IVa: 28 patients (50.9%); Stage IVb: 11 patients (20%); and Stage IVc: two patients (3.6%). Overall, only nine patients (16.4%) were diagnosed at an early stage, whereas 46 patients (83.6%) were diagnosed with advanced disease. Lymph node metastasis was observed in 25 patients (45.5%), and while 30 patients (54.5%) were classified as N0 (node-negative).

### 3.3. Risk Factors

A total of 32 patients were smokers, six of whom continue to smoke post-diagnosis. The mean tobacco index was 6.7 ± 12.5 pack-years. A total of 34 patients reported alcohol consumption, with six patients continuing to consume alcohol after diagnosis. A total of 26 cases had a history of periodontal disease. The use of dental prostheses was documented in 10 patients, of whom six had fixed prostheses and four had removable prostheses.

HPV was detected in only three patients, representing 5.4% of the sample; among these, only one sample was positive for P16, as determined by immunohistochemistry. APOBEC-related signatures were identified in 67% of the patients, whereas no microsatellite instability (MSI) signatures were detected. EBV was detected in 15 patients, representing 27.3% of the cohort; among these, of which 40% exhibited APOBEC-related mutational signatures, and 6.6% had microsatellite instability signatures. Neither HPV nor EBV was associated with prognostic indicators.

### 3.4. Histopathological Data

The average DOI was measured 8.5 mm (range: 0.3–25 mm), with assessments revealing lymphovascular invasion in 21 patients (38%) and perineural invasion in 18 patients (32.7%). Histological grading showed moderate differentiation in 35 patients (63.6%), well-differentiated tumors in 8 patients (14.5%), and poorly differentiated tumors in 11 patients (20%). A total of 38 patients underwent the surgery. Surgical margins included negative margins in 7 patients (18.4%), positive margins in 14 patients (36.8%), and close margins in 17 patients (44.7%). Tumor dissemination and invasion patterns revealed that the highest number of patients exhibited patterns 4 and 5, respectively, with 15 patients (27.3%) in each category.

### 3.5. Prognostic Data

Disease recurrence was observed in eight patients, with a median time to recurrence of 26.3 months. Four patients showed a complete response to treatment with radical radiotherapy or chemoradiotherapy, whereas stable disease progression occurred in 10 patients, eventually leading to death. At the end the follow-up, 29 patients were alive, while 26 patients died. Early-stage mean overall survival (OS) was 57.2 months (95% CI: 43.6–70.7), with mean survival of 78% at 24 months. The median advanced-stage OS was 25.5 months (95% CI: 0–72.1), with median survival of 44% at 24 months. Early-stage mean disease-free survival (DFS) was 48.1 months (95% CI: 29.5–66.7), with 67% of patients still disease-free at 24 months. The advanced-stage mean DFS was 63.9 months (95% CI: 56.3–71.6), with 81% of the patients still disease-free at 24 months.

### 3.6. Identification of Pathogenic Variants

**Figure 1:**
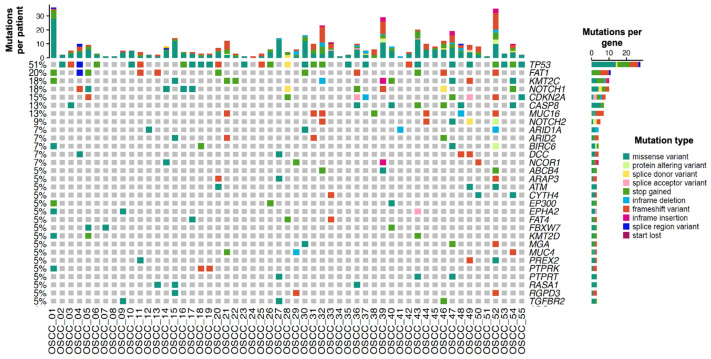
Genetic alterations in Mexican patients with oral squamous cell carcinoma. Oncoprint depicting the nucleotide variants as mutation types present in three patients or more. The upper graph represents the total number of mutations per patient, while the right graph bar represents the total number of patients with mutations in each gene. Each mutation type is color-coded according to the Mutation type legend.

A total of 274 mutations were identified, with a maximum of 36 mutations and a minimum of one mutation per patient (Figure 1 and Figure S1). The pathogenic variants detected in at least 7% of patients with OSCC involved 13 altered genes: *TP53* mutations were detected in 51% of patients, with 45.4% being missense mutations. *FAT1* mutations were detected in 20% of the patients, of which 50% were stop-gain mutations and 41.6% were frameshift mutations. *KMT2C* mutations were detected in 18% of the patients, of which 42.8% were stop-gain mutations. *NOTCH1* mutations were present in 18% of patients and consisted of stop-gain, missense, and splice donor mutations. *CDKN2A* mutations were identified in 15% of the patients and including stop-gain mutations, frameshift mutations, and splice acceptor mutations. *CASP8* mutations were found in 13% of the patients, of which 71.4% were missense mutations. *MUC16* mutations were also found in 13% of the patients and consisted predominantly of frameshift mutations. *NOTCH2* mutations were detected in 9% of the patients. Additionally, 7% of the patients carried the following mutated genes: *ARID1A*, *ARID2*, *BIRC6*, *DCC*, and *NCOR1* (Table S2).

### 3.7. Identification of Signaling Pathways

A total of 67 signaling pathways were affected. The 10 most frequently altered pathways were the JAK/STAT signaling pathway (with an enrichment score of 17.2), glucose deprivation-induced p53 pathway (13.3), insulin/IGF-activated MAP kinase/protein kinase cascade (13.3), p53 feedback loop circuit 1 (10.4), axon guidance mediated by semaphorins (9.9), p53 pathway (9.1), PDGF pathway (9.1), axon guidance mediated by Slit/Robo (8.7), angiogenesis (7.8), and protein kinase B cascade in the insulin/IGF signaling pathway (7.5). A complete list of the signaling pathways is present in Table 3. The NOTCH pathway was the most frequently affected by non-smokers and non-drinkers. In patients with the SBS 5 mutational signature, the most frequently altered pathway was the p53 feedback loop circuit, whereas in patients with the ID-19 mutational signature, the glucose deprivation-induced p53 pathway was the most affected.

### 3.8. Tumor Mutational Burden (TMB)

**Figure 2:**
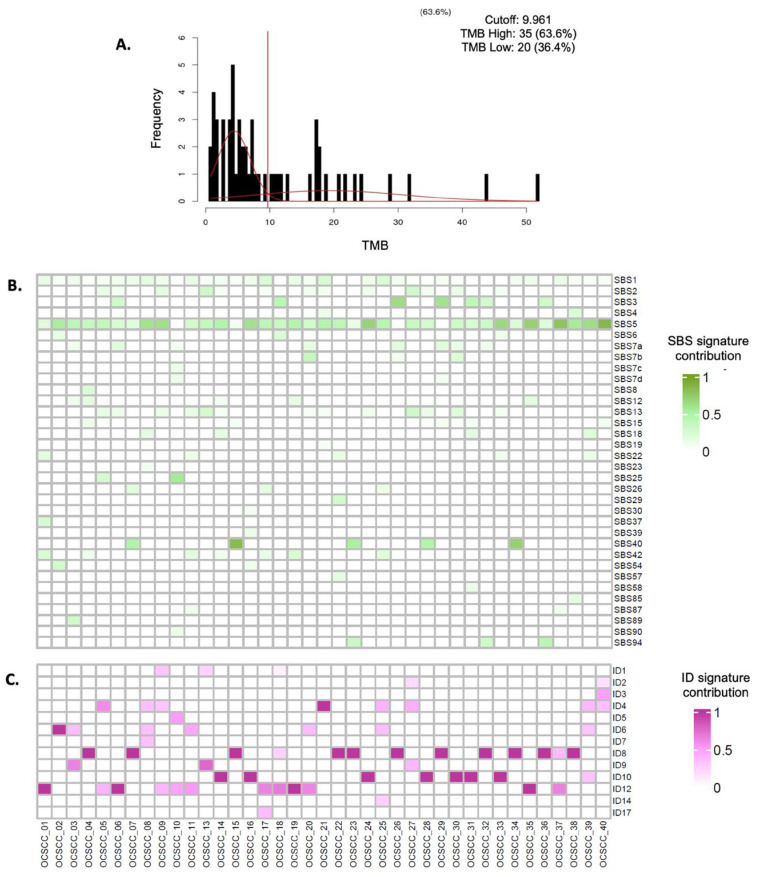
Integrated analysis of tumor mutation burden and mutational signatures (SNVs and indels). (**A**). Mixture model adjustment using CutoffFinder V1 (A two-Gaussian distribution mixture model was fitted to the biomarker histogram using CutoffFinder V1 software. The optimal cutoff is determined as the point where the probability density functions of the mixed distribution intersect, indicated by the vertical line marking the cutoff point. (**B**). Single-base substitution (SBS) mutational signatures identified in Mexican patients with oral squamous cell carcinoma. The color intensity represents the contribution of the SBS 5 signature in each patient, with darker shades indicating a higher contribution. (**C**). Insertion–deletion (InDel) mutational signatures found in Mexican patients with oral squamous cell carcinoma. The intensity of the color represents the contribution of the SBS 5 signature in each patient: the darker the color, the higher the contribution.

Regarding the total number of somatic mutations found in the DNA of patients with OSCC, the median TMB was determined to be 10.47 mutations per megabase (mut/Mb). The highest recorded mutation rate was 51.75 mut/Mb, whereas the lowest was 0.8 mut/Mb. For analysis and correlation with survival, TMB was categorized as high, corresponding to 20 patients (36.4%), and low, corresponding to 35 patients (63.6%) (Figure 2A).

### 3.9. Mutational Signatures

The most prevalent signature was SBS 5, detected in 24 patients (43.6%), followed by SBS 1 in 12 patients (21.8%), SBS 13 in nine patients (16.4%), SBS 12 and SBS 37 in eight patients each (14.5%), SBS 3, SBS 7a, and SBS 40 in six patients each (10.9%), and SBS 15, SBS 22a, and SBS 96 in five patients each (9.1%) (Figure 2B and Table S3). The most represented InDel signature was ID-19, present in 19 patients (34.5%), followed by ID-08 in eight patients (14.5%), ID-12 in seven patients (12.7%), ID-06 and ID-10 in six patients each (10.9%), and ID-01, ID-02, and ID-04 in five patients each (9.1%) (Figure 2C and Table S4). Smoking-related signatures (SBS 4 and SBS 5, likely) were found in 28 patients (50.9%), microsatellite instability signatures (SBS 3, 6, 15, and 26) in 11 patients (20%), APOBEC enzyme and inflammation signatures (SBS 2 and 13) in nine patients (16.3%), and ultraviolet light exposure signatures (SBS 7a, b, c, d, and SBS 38) in eight patients (14.5%).

### 3.10. Clinical–Molecular Associations

#### 3.10.1. Mutations

*TP53* mutations were associated with the presence of metastatic lymph nodes (n = 19, 67.9% vs. n = 6, 22.2%; *p* = <0.001), disease recurrence (n = 7, 25% vs. n = 1, 3.7%; *p* = 0.025), disease persistence (n = 10, 35.7% vs. n = 3, 11.1%; *p* = 0.032), and disease progression (n = 10, 35.7% vs. n = 2, 7.4%; *p* = 0.011). *TP53* was strongly correlated with the presence of lymph node metastases, as demonstrated by logistic regression analysis, with an odds ratio (OR) of 7.3 (95% CI: 2.2–24.6, *p* = 0.001; Nagelkerke’s R^2^ = 0.262). The association was also confirmed using the chi-square test (odds ratio (OR): 7.3; 95% CI: 2.2–24.6, *p* = <0.001).

The presence of *FAT1* mutations was associated with tumor spread (WPOI-5) (n = 7, 63.6% vs. n = 8, 18.2%; *p* = 0.005). This association was further confirmed through logistic regression analysis (OR = 7.8, 95% CI: 1.8–33.4, *p* = 0.005), with a Nagelkerke R^2^ value of 0.222. Additionally, the chi-square analysis with Fisher’s adjustment corroborated this relationship (OR = 7.8, 95% CI: 1.8–33.5, *p* = 0.005). *NOTCH1* mutation was associated with the presence of the mutational signature SBS 4 (n = 3, 30% vs. n = 1, 2.2%; *p* = 0.016). *KMT2C* mutation was associated with high tumor mutational burden (TMB) (n = 8, 80% vs. n = 12, 26.7%; *p* = 0.003), mutational signature ID-19 (n = 7, 70% vs. n = 12, 26.7%; *p* = 0.023), and the mutational signature SBS 15 (n = 3, 60% vs. n = 2, 4.4%; *p* = 0.037). *MUC16* mutations were associated with lower prevalence of tobacco-related mutational signatures (n = 1, 14.3% vs. n = 27, 56.3%; *p* = 0.045) and mutational signature ID-19 (n = 7, 100% vs. n = 12, 25%; *p* < 0.001). *NOTCH2* mutations were associated with microsatellite instability-related mutational signatures (n = 3, 60% vs. n = 8, 16%; *p* = 0.049) and mutational signature ID-19 (n = 5, 100% vs. n = 14, 28%; *p* = 0.003).

#### 3.10.2. Patients with No Exposure to Tobacco and Alcohol

Among the patients with no classical risk factors for oral cancer (n = 17, 30.9%), the majority were female (n = 14, 82.4% vs. n = 11, 28.9%) in the smoking and/or alcohol consumption group (*p* < 0.001). The presence of *CYTH4* mutation was significantly higher in the exposed group (n = 3, 17.6% vs. n = 0) in the exposed group (*p* = 0.026). Furthermore, patients in this group had an ID-19 mutational signature (n = 10 cases, 58.8% vs. n = 9 cases, 23.7%; *p* = 0.011).

#### 3.10.3. Tumor Mutational Burden (TMB) and Its Clinical Correlations

High TMB was associated with periodontal disease (n = 13, 65%; *p* = 0.002), *KMT2C* mutation (n = 8, 40% vs. n = 2, 5.7%; *p* = 0.003), *MUC16* and *CASP8* mutations (n = 6, 30% each vs. n = 1, 2.9%; *p* = 0.007), and the presence of the ID-19 mutational signature (n = 13, 65% vs. n = 6, 17.1%; *p* < 0.001); conversely, and low TMB was associated with tobacco-related mutational signatures (n = 22, 62.9% vs. n = 6, 30%; *p* = 0.019) and the presence of the SBS 5 signature (n = 19, 54.3% vs. n = 5, 25%; *p* = 0.035).

#### 3.10.4. Mutational Signatures and Prognostic Implications

The SBS 5 signature was associated with the presence of periodontal disease (n = 8, 33.3% vs. n = 2, 8.3%; *p* = 0.004), higher recurrence risk (OR = 12.3, 95% CI: 1.3–109; *p* = 0.016), lower tumor burden (OR = 3.5, 95% CI: 1–11.9, *p* = 0.035), and absence of the *MUC16* mutation (*p* = 0.015). The ID-19 mutational signature was also associated with similar factors: patients with no exposure to tobacco and alcohol (n = 10, 52.6% vs. n = 7, 19.4%; *p* = 0.011), presence of periodontal disease (n = 11, 57.9% vs. n = 15, 41.7%; *p* = 0.001), presence of *KMT2C* (OR = 6.4, 95% CI: 1.4–28.9; *p* = 0.023), *MUC16* (n = 7, 36.8% vs. n = 0; *p* = <0.001), and *NOTCH2* (n = 5, 26.3% vs. n = 0; *p* = 0.003) mutations, and higher tumor mutational burden (TMB) (OR = 8.9, 95% CI: 2.5–32, *p* < 0.001). The ID-19 mutational signature was associated with a lower prevalence of SBS5 and tobacco-related signatures (n = 3, 15.8% vs. n = 21, 58.3%; *p* = 0.002 and n = 6, 31.6% vs. n = 22, 61.1%; *p* = 0.037, respectively). The clinical correlations of these additional signatures are detailed in Tables S3 and S4.

### 3.11. Survival Analysis

#### 3.11.1. Overall Survival

Molecular alterations with a significant impact on OS included the presence of the *TP53* mutation, as determined by Kaplan–Meier analysis, with a median OS of 15.4 months compared with 54.2 months in its absence (*p* = 0.019). Additionally, the *FAT1* mutation demonstrated a significant impact based on Cox regression analysis, with an HR of 3.1 (95% CI: 1.1–8.6, *p* = 0.027).

**Figure 3:**
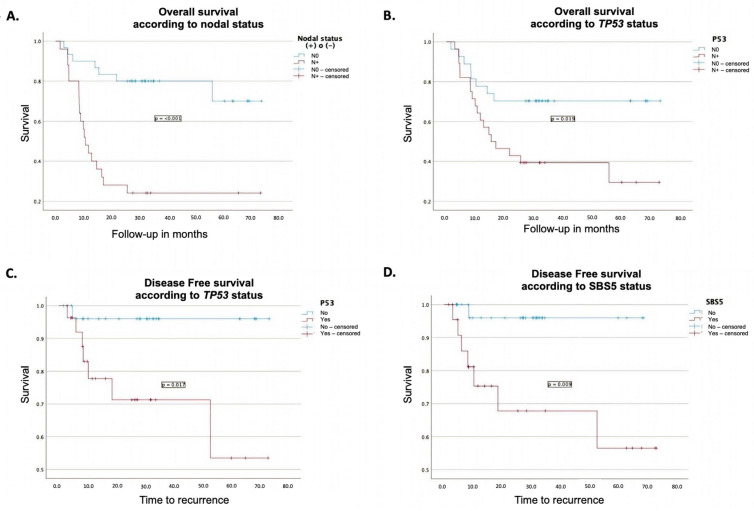
Prognostic impact of clinical and molecular features on survival outcomes. (**A**). Impact of lymph node status on overall survival measured using the Kaplan–Meier method. (**B**). Impact of TP53 mutation on overall survival measured using the Kaplan–Meier method. (**C**). Kaplan–Meier curve showing the impact of TP53 mutation on disease-free survival. (**D**). Kaplan–Meier curve showing the effect of SBS 5 on disease-free survival.

The presence of lymph node metastases had a pronounced effect on survival in both Kaplan–Meier and Cox regression analyses. Patients with lymph node metastases had a median OS of 10.5 months compared with 59.1 months in those without metastases. The corresponding HR was 6.9 (95% CI: 2.3–21, *p* < 0.001) in both analyses. Further details on the survival outcomes are presented in Table 4 and Table 5, and Figure 3.

#### 3.11.2. Disease-Free Survival

The presence of *TP53* mutations was associated with worse disease-free survival (DFS) in both Kaplan–Meier and Cox regression analyses. Patients harboring this mutation had a mean DFS of 51.3 months, compared to 70.6 months for those without the mutation (*p* = 0.017). Furthermore, in Cox regression analysis, *TP53* mutations showed an HR of 11.5 (95% CI: 1.3–99.9, *p* = 0.027) (Table 4 and Table 5 and Figure 3).

Similarly, the presence of the SBS5 mutational signature was associated with poorer DFS in both Kaplan–Meier and Cox regression analyses. Patients with this signature had a mean DFS of 50.4 months, compared with 66.5 months for those without the signature (*p* = 0.009). In the Cox regression model, the SBS5 signature was associated with an HR of 11.1 (95% CI: 1.1–101.1, *p* = 0.032) (Table 4 and Table 5 and Figure 3).

## 4. Discussion

OSCC remains a significant oncologic challenge, with high morbidity and mortality rates, particularly in developing regions. In Mexico, OSCC accounts for approximately 2% of malignant neoplasms, yet 70% of patients present with locally advanced disease at diagnosis, leading to reduced treatment efficacy and a five-year survival rate of approximately 50% [2,30,31]. This underscores the need for novel biomarkers to facilitate early detection, improve prognostic stratification, and guide targeted therapy. In this study, we performed WES of fresh tumor tissue to analyze pathogenic variants, mutational signatures, and tumor mutational burden (TMB), and assessed their clinical implications [48].

Demographically, 31% of our patients did not present classical risk factors for this cancer, such as tobacco and alcohol exposure, suggesting alternative carcinogenic pathways, including chronic inflammation and viral infections. This subset of patients, particularly non-smoking females, exhibited a distinct molecular profile and represents an emerging clinical phenotype that warrants further investigation. Consistent with global trends, most tumors arise on the mobile tongue [16] and are diagnosed at advanced stages (III–IV), which correlates with increased lymph node involvement, poorer prognosis, and reduced overall survival. Only 16% of cases were detected at an early stage; three of these early-stage patients experienced tumor recurrence within one year, and two subsequently died. These tumors showed deep invasion, *TP53* mutations, and low TMB, suggesting that conventional staging alone may be insufficient for accurate risk stratification [49]. Our findings support the incorporation of molecular biomarkers into clinical decision-making to improve prognostic precision and guide individualized treatment [50].

Interestingly, no association was observed between survival and traditional risk factors, including tobacco use, even though 58% of patients had a history of smoking. Reported smoking prevalence in White populations ranges from 30% to 96% [25,26,27,28,29,30,31,32,33,34,35,36,37,38,39,40,51]. In contrast, none of our patients consumed areca or betel nuts, which are recognized as major risk factors for OSCC in Eastern populations [4,35]. Alcohol consumption was reported by 38% of the patients, mostly at moderate levels, with only one study documenting a higher prevalence (89%) [32,52]. Despite its direct and synergistic carcinogenic effects on tobacco [4,9,15], alcohol consumption was not associated with prognosis in this study [40]. The HPV prevalence observed in our study is consistent with previous reports in Western populations and is significantly lower than that found in oropharyngeal squamous cell carcinoma (OPSCC) [10,11,12]. EBV prevalence (27%) also agreed with previous studies [12,13,14]. Tumors harboring HPV or EBV infections exhibited a higher prevalence of APOBEC-related mutational signatures, which is consistent with viral-induced DNA damage, although no significant correlation with clinical outcomes was observed.

*TP53* mutations were the most frequent alterations, with a prevalence like those observed in Asian cohorts but lower than those observed in White populations (TCGA data) [25,26,27,28,29,30,31,32,33,34,35,36,37,38,39,40]. *TP53* encodes a tumor suppressor protein that is involved in DNA repair, cell cycle regulation, apoptosis, and genomic stability. *TP53* mutations were also associated with lymph node metastases and significantly worse OS and DFS. This suggests that nodal involvement may act as a practical histological surrogate for TP53-driven tumor aggressiveness. In clinical practice, *TP53* status could support the choice of more radical surgery, justify adjuvant CT and/or CRT even in early-stage cases, and indicate the need for closer surveillance to enable the early detection of recurrence. *FAT1* mutations an also common and include a high proportion of stop-gain variants predicted to yield truncated proteins. Loss of *FAT1*, which encodes a cadherin-family adhesion molecule, may promote epithelial–mesenchymal transition and enhance invasiveness and therapy resistance [25,26,27,28,29,30,31,32,33,34,35,36,37,38,39,40]. In our cohort, *FAT1* mutations were associated with the worst pattern of invasion-5 (WPOI-5), which might aid in the risk stratification and selection of patients for intensified local control. *KMT2C* mutations were identified in 18% of cases, a rate higher than that reported in TCGA [29]. Because *KMT2C* encodes a histone methyltransferase that regulates transcription, its mutation can drive epigenetic dysregulation and heighten immune responses [47]. In our data, *KMT2C* mutations were correlated with high TMB, as well as SBS15 and ID-19 signatures, suggesting a potentially favorable prognosis and greater likelihood of response to immune checkpoint inhibitors [48,49,50,51,52], supporting their use as predictive biomarkers for immunotherapy.

Mutations in *NOTCH1*, *CDKN2A*, *CASP8*, *MUC1*, and *NOTCH2*, which are involved in epithelial growth factor signaling, RAS pathway regulation, apoptosis, and mucosal protection, occurred at frequencies like those reported in previous studies [25,26,27,28,29,30,31,32,33,34,35,36,37,38,39,40]. Additionally, several mutations not previously reported in OSCC sequencing studies were detected in 7% of cases including mutations in *ARID1A* (chromatin remodeling), *BIRC6* (apoptosis inhibition), *DCC* (neural regulation and tumor suppression), and *NCOR1* (transcriptional inhibition) [13,14,15,16,19,20,21,22,23,24,25]. Another gene with the same prevalence, *ARID2*, was previously reported in a Pakistani cohort [35] and is involved in the regulation of gene transcription, cellular differentiation, and proliferation, further emphasizing the relevance of chromatin remodeling alterations in OSCC. These findings suggest that OSCC progression is influenced by a combination of well-characterized oncogenic pathways (e.g., RAS and apoptosis dysregulation) and epigenetic modifications involving chromatin remodeling factors. To contextualize the genomic findings in our Mexican OSCC cohort, we performed a comparative analysis of the mutational frequencies reported in other populations, including Caucasian, Asian, Indian, and Arab cohorts, based on previously published genomic studies. We used data from TCGA as our principal reference, given its robust methodology and status as the most comprehensive and standardized genomic dataset for head and neck squamous cell carcinomas which are primarily from Caucasian individuals. Additionally, we performed comparisons with other international cohorts reported in the recent literature to identify molecular patterns that are either shared across populations or potentially unique to the Mexican cohort. This comparative analysis is detailed in Supplementary Table S2 and highlights the population-specific differences in mutation prevalence and signature distribution. Pathway analysis revealed frequent alterations in JAK/STAT signaling, p53 signaling under glucose deprivation, and the insulin/IGF–MAPK cascade, in contrast to the NOTCH, RTK/RAS/MAPK, and TGF-β which were emphasized in the only previous pathway-level OSCC study [22]. This discrepancy may reflect population-specific risk factors, such as betel nut exposure in Taiwanese cohorts. The distinct molecular landscape observed in this study highlights the need for population-tailored genomic profiling to refine biomarker-driven therapeutic strategies.

Several well-characterized SBS mutational signatures have been identified in this cohort, including SBS 12, 37, 40a, 22a, and 96 [13,23,26]. Nearly half of the patients (43.6%) exhibited the SBS5 signature, which is linked to aging, smoking, and DNA-repair defects. In our cohort, SBS5 was correlated with periodontal disease, lower TMB, higher recurrence risk, and absence of MUC16 mutations. Tumors with SBS5 also showed preferential activation of p53 feedback, NOTCH1 signaling, angiogenesis, and PDGF pathways, differing from the MAPK and axonal-guidance pathways that are predominant in SBS5-negative tumors. These findings suggest that SBS5 is a biologically distinct, clinically aggressive OSCC subtype and may serve as a biomarker for poor prognosis and limited response to immunotherapy [26]. Our study suggests that chronic inflammation and dysbiosis may contribute to carcinogenesis, influencing tumor immunogenicity and response to immunotherapy. Although SBS 5 lacks a well-defined etiology, it has been proposed to be tobacco-related due to its association with bladder cancer [27]. Microsatellite instability (MSI)-related signatures a strongly associated with periodontal disease and high TMB, reinforcing their potential as indicators of favorable prognosis and immunotherapy responsiveness [50,51,52]. APOBEC signatures, which are more common in HPV- or EBV-positive tumors, further underscore the role of virus-driven mutagenesis; however, no significant association with dental prosthesis use or periodontal disease was identified. There is limited literature on the InDel signatures in OSCC. We identified several previously unreported signatures, with ID-04, 06, 08, 10, 12, and 19 being the most prevalent. Notably, ID-19 was uniquely associated with non-smoking and non-drinking status, periodontal disease, and high TMB, suggesting that periodontal disease may be a potential mutational driver, although the biological process underlying this signature remains unknown [29,45]. Because ID-19 is correlated with high TMB, it may indicate tumors with favorable prognoses and potential sensitivity to immune checkpoint inhibition [40]. ID-19-positive tumors showed distinct signaling features, including p53 signaling under glucose deprivation, whereas ID-19-negative tumors were enriched for JAK/STAT activation, implying divergent oncogenic trajectories compared with other OSCC subtypes. SBS5 and ID-19 a strongly correlated with periodontal disease, linking chronic inflammation and microbiome dysbiosis to OSCC development. These signatures may support periodontal disease prevention as a cancer control strategy and could serve as early molecular markers in premalignant lesions, aiding in early detection and risk stratification.

TMB analysis revealed that low TMB correlated with tobacco-related SBS4 and SBS5, whereas high TMB was associated with periodontal disease and mutations in *KMT2C*, *MUC16*, and *CASP8*, as well as the ID-19 signature. Although previous studies across multiple malignancies have suggested that high TMB is often associated with MSI [25], leading to increased tumor neoantigen presentation, enhanced immune recognition, and improved prognosis [50,51,52], we did not observe a significant impact on survival outcomes, suggesting that additional molecular factors modulate the prognostic value of TMB in OSCC. A focused analysis of the subgroup of patients with OSCC who did not consume tobacco or alcohol (31% of the cohort) revealed a predominance of young women diagnosed with advanced clinical stages, suggesting a distinct etiologic and molecular subgroup. These patients exhibited a higher prevalence of the ID-19 signature and a unique mutation in CYTH4, a gene not previously reported in OSCC sequencing studies [13,14,15,16,19,20,21,22,23]. CYTH4, which regulates cell adhesion, migration, and immune responses, may promote tumor progression by enhancing motility and immune evasion. The concurrent enrichment of NOTCH signaling in this subgroup further supports a distinct oncogenic pathway that warrants further investigation.

A major strength of this study lies in its comprehensive WES analysis of OSCC across all anatomical subsites and clinical stages in a Mexican cohort, which is an underrepresented population in cancer genomics. The inclusion of patients with and without traditional risk factors enabled the discovery of novel molecular signatures (ID-19, SBS5) and candidate genes (*CYTH4* and *KMT2C*) with potential clinical utility. Viral integration analysis, proteomic profiling, and immune cell characterization were not performed, thus limiting the mechanistic interpretation of the observed associations. Although our cohort of 55 patients was relatively small, it represents the largest exome-sequenced series of Mexican squamous cell carcinomas reported to date. The single-center design may introduce selection bias and limit the generalizability of the findings, and we recognize that multivariate analyses performed with this sample size should be considered exploration and hypothesis-generating. To minimize overfitting, variables were included in the models only when supported by clinical or statistical relevance, and in accordance with the recommended event-to-covariate ratios. Despite these inherent limitations, this study provides valuable preliminary insights into the molecular landscape of Mexican OSCC and its clinical correlations, thus offering a foundation for future research. Larger multicenter cohorts with independent validation will be essential to confirm the mutational signatures and candidate biomarkers identified here. Furthermore, functional characterization of the detected mutations is beyond the scope of this pilot study. We recognize this as a critical component and therefore plan to address it in subsequent studies using representative in vivo and in vitro systems, such as patient-derived lines, organoids or xenograft models, to clarify the biological significance of key alterations, particularly the activation of the CYTH4 and NOTCH pathways, which, to the best of our knowledge, have not been previously reported in OSCC.

This exploratory study was not designed to validate the biomarkers. Prospective multicenter studies are needed to confirm the clinical utility of alterations in *TP53*, *FAT1*, *KMT2C*, SBS5, and ID-19 in diverse populations. These biomarker associations require external validation before clinical implementation. The integration of WES with liquid biopsy technologies, including circulating tumor DNA, exosomal RNA, and microRNAs, may enhance the early molecular detection and monitoring of OSCC. A combined model that incorporates TNM staging with genomic biomarkers may improve prognosis and treatment selection. Our findings were systematically compared with international genomic datasets, with TCGA serving as the primary reference cohort owing to its scale, data quality, and extensive use in OSCC research. While informative, we acknowledge the limitations posed by the lack of publicly available genomic data from Latin American and other underrepresented populations. Therefore, we emphasize the urgent need for multicenter genomic studies that include ethnically diverse cohorts, particularly from regions such as Latin America, to better understand the molecular heterogeneity of OSCC and to advance equitable precision oncology strategies. Although several of the driver mutations we identified have been reported in other populations, our study is the first to document their prevalence and specific clinical associations—such as the strong link with periodontal disease—within a Mexican OSCC cohort. Moreover, the discovery of the previously unreported ID-19 InDel signature underscores a distinct mutational process not described in other ethnic groups, highlighting population-specific molecular features that expand the current knowledge beyond what has been observed in non-Mexican cohorts. Further characterization of OSCC in non-smoking, non-drinking patients—especially young women—could reveal alternative oncogenic mechanisms involving NOTCH signaling, immune evasion, and epigenetic regulation. Longitudinal studies of the oral microbiome and inflammatory mediators are needed to clarify the role of chronic inflammation and periodontal disease, particularly in tumors harboring SBS5 and ID-19. Finally, evaluation of *KMT2C* mutations, MSI-related signatures, and high TMB as predictors of immunotherapy response, as well as assessment of *TP53* and *FAT1* mutations as markers of treatment resistance and aggressive disease, may refine clinical decision-making and support personalized therapy.

## 5. Conclusions

Collectively, our findings provide critical insights into OSCC pathogenesis and prognosis, supporting the integration of molecular profiling into routine clinical practice to improve early detection, prognostic accuracy, and personalized therapy selection. These findings confirmed the significance of lymph node metastases and clinical stage as key prognostic factors. Furthermore, they highlighted population-specific molecular differences and suggested alternative oncogenic pathways beyond classical risk factors. The strong association between periodontal disease, high TMB, and specific mutational signatures (SBS 5, ID-19, and MSI-related signatures) suggests that chronic inflammation and oral microbiome dysbiosis play an underappreciated role in OSCC pathogenesis. Both *TP53* and *FAT1* mutations were linked to nodal metastases and WPOI-5, suggesting their potential as histological surrogates for aggressive tumor behavior. *KMT2C* mutations were correlated with high TMB, suggesting that KMT2C-mutant OSCC may be more immunogenic and responsive to immune checkpoint inhibitors. ID-19, an InDel signature not previously reported in OSCC, emerged as a defining feature in 35% of cases, particularly among non-smoking and non-drinking patients. Its association with high TMB and unique signaling pathways indicates an inflammation-driven carcinogenic mechanism. These findings support ID-19 as a novel prognostic and predictive biomarker for OSCC, particularly in patients lacking traditional risk factors. The identification of HPV and EBV infections suggests the involvement of alternative carcinogenic mechanisms, including chronic inflammation, viral infections, and epigenetic dysregulation, which warrant further investigation.

## Figures and Tables

**Table 1:** Demographic characteristics and risk factors of the patients (N = 55).

Characteristic		n	%
Age (mean years; Range)		61.3 (21–86)	NA
Follow up (media months; Range)		27.9 (1.6–73.4)	NA
Sex			
	Female	25	45.5
Periodontal disease			
	Yes	26	47.3
Use of dental prosthesis			
	Fixed	6	10.9
	Removable	4	7.3
	No prosthesis	45	81.8
Smoking status (Tobacco index: 6.7, SD 12.5)			
	Yes	32	58.2
Alcohol consumption			
	Yes	34	61.8
Alcohol intake severity			
	Mild	13	23.6
	Moderate	15	27.3
	Intense	7	12.7
Clinical stage			
	0	1	1.8
	I	3	5.5
	II	5	9.1
	III	5	9.1
	Iva	28	50.9
	IVb	11	20.0
	IVc	2	3.6
Categorized Stage			
	Early	9	16.4
	Advanced	46	83.6
Response to neoadjuvant treatment			
	Partial response	4	7.3
	Stable disease or progression	10	18.2
	No neoadjuvant treatment	41	74.5
Recurrence			
	Yes	8	14.5

NA: not applicable.

**Table 2:** Clinicopathological characteristics of the patients (N = 55).

Characteristic		n	%
Histological grade			
	Well differentiated	8	14.5
	Moderately differentiated	35	63.6
	Poorly differentiated	11	20.0
	Not assessable	1	1.8
Invasion pattern			
	Pattern 1	0	0.0
	Pattern 2	4	7.3
	Pattern 3	4	7.3
	Pattern 4	15	27.3
	Pattern 5 (WPOI-5)	15	27.3
	Not assessable	17	30.1
Depth of invasion (mean mm; range)		8.5 (0.3–25)	
Lymphovascular invasion			
	Yes	21	38.2
Perineural Invasion			
	Yes	18	32.7
AJCC subsite			
	Mobile tongue	25	45.5
	Lower gingiva	6	10.9
	Upper gingiva	10	18.2
	Floor of the mouth	4	7.3
	Buccal mucosa	1	1.8
	Hard palate	4	7.3
	Retromolar trigone	5	9.1
Tumor size			
	Tis	1	1.8
	T1	3	5.5
	T2	8	14.5
	T3	5	9.1
	T4a	32	58.2
	T4b	6	10.9
Lymph node involvement			
	N0	30	54.5
	N1	4	7.27
	N2a	3	5.5
	N2b	4	7.3
	N2c	5	9.1
	N3a	0	0.0
	N3b	9	16.4
Treatment			
	Surgery + RT	12	21.2
	CRT	8	14.5
	Induction CT + CRT	1	1.8
	CRT + Surgery	1	1.8
	Surgery + CRT	8	14.5
	RT	4	7.3
	Palliative (Surgery, RT, CT)	3	5.5
	No treatment initiated	18	32.7
Surgical margins			
	Negative	7	12.7
	Close	17	30.9
	Positive	14	25.5
	No surgery	17	30.9
Sarcomatous component			
	Yes	7	12.72
Overall survival			
	Early stage	57.2 months (95% CI: 43.6–70.7), 78% at 24 months	
	Advanced stage	25.5 months (95% CI: 0–72.1), 44% at 24 months	
Disease-free survival			
	Early stage	48.1 months (95% CI: 29.5–66.7), 67% at 24 months	
	Advanced stage	63.9 months (95% CI: 56.3–71.6), 81% at 24 months	

RT: radiotherapy; CRT: chemoradiotherapy; CT: chemotherapy; CI: confidence interval; Tis: carcinoma in situ; WPOI-5: worst pattern of invasion classification.

**Table 3:** Altered signaling pathways in patients with oral squamous cell carcinoma.

Pathway	No. of Genes in the Pathway/No. of Genes Identified	Enrichment Score
JAK/STAT signaling pathway	17/4	17.2
Glucose deprivation-induced p53 pathway	22/4	13.3
Insulin/IGF-MAP kinase/protein kinase cascade	33/6	13.3
p53 feedback loop circuit 1	7/1	10.4
Axon guidance mediated by semaphorins	22/3	9.9
p53 pathway	88/11	9.1
PDGF signaling pathway	144/18	9.1
Axon guidance mediated by Slit/Robo	25/3	8.7
Angiogenesis	169/18	7.8
Protein kinase B cascade in the insulin/IGF pathway	39/4	7.5

**Table 4:** Analysis of overall survival (OS) and disease-free survival (DFS) using Kaplan–Meier and Cox regression.

Overall Survival (OS)				Disease-Free Survival (DFS)			
Variable	Kaplan–Meier (Months)	*p*-Value	Cox Regression (HR, 95% CI, *p*-Value)	Variable	Kaplan–Meier (Months)	*p*-Value	Cox Regression (HR, 95% CI, *p*-Value)
*TP53*	15.4 vs. 54.2	0.019	---	*TP53*	51.3 vs. 70.6	0.017	11.5 (1.2–99.9) *p* = 0.027
*FAT1*	---	---	3.1 (1.1–8.6) *p* = 0.027	*FAT1*	---	---	---
Lymph node metastases	10.5 vs. 59.1	<0.001	6.9 (2.3–21) *p* < 0.001	Lymph node metastases	---	---	---
SBS 5 signature	---	---	---	SBS 5 signature	50.4 vs. 66.5	0.009	11.1 (1.1–101.1) *p* = 0.032

HR: Hazard Ratio.

**Table 5:** Cox regression model for overall survival (OS) and disease-free survival (DFS).

**Overall Survival**				
**Variable**	***p*-Value**	**Hazard Ratio (HR)**	**95% CI (Lower Bound)**	**95% CI (Upper Bound)**
*FAT1* Mutation	0.027	3.1	1.1	8.6
Lymph Node Status	<0.001	6.9	2.3	21
Low Tumor Mutational Burden (TMB)	0.309	1.7	0.6	4.9
*TP53* Mutation	0.378	1.5	0.5	4.1
*CDKN2A* Mutation	0.035	0.2	0.06	0.9
*KMT2C* Mutation	0.069	3.3	0.9	12.7
Model *p*-value = <0.001				
**Disease-Free Survival (DFS)**				
**Variable**	***p*** **-Value**	**Hazard Ratio (HR)**	**95% CI (Lower Bound)**	**95% CI (Upper Bound)**
SBS 5 Signature	0.032	11.1	1.2	101.1
*TP53* Mutation	0.027	11.5	1.3	99.9
Tumor Invasion Pattern (WPOI-5)	0.216	3.2	0.4	21.6
*CDKN2A* Mutation	0.983	0	0	--
Model *p*-value = 0.001				

## Data Availability

All relevant data are available from the corresponding authors upon reasonable request.

## Supplementary Materials

The following supporting information can be downloaded at: https://www.mdpi.com/article/10.3390/cancers17203282/s1, Figure S1. Genetic alterations in Mexican patients with oral squamous cell carcinoma. Table S1. Summary of Major Sequencing Studies in Oral Cancer; Table S2. Main Functions of Proteins in Pathogenic Variants Identified in OSCC Sequencing Analysis; Table S3. Single Base Substitution (SBS) Signatures in Oral Squamous Cell Carcinoma (OSCC); Table S4. Insertion and Deletion (InDel) Signatures.
