# Supplementary material for: Molecular Characterization of Oral Squamous Cell Carcinoma in Mexican Patients: A Genomic and Epidemiological Overview
Source: Cancers (Basel). 2025 Oct 10;17(20):3282. doi: 10.3390/cancers17203282 (PMC12564337; doi:10.3390/cancers17203282)
Supplement: Supplementary file 1 [file cancers-17-03282-s001.zip › Table S1.pdf]

Table S1. Summary of Major Sequencing Studies in Oral Cancer

Author	Patients (n)	Population	Risk factors	Subsites	Mutated genes	Additional data
**Agrawal et al. (2011)**	25*	White	Tobacco 76%	—	*TP53, NOTCH1, CDKN2A, PIK3CA, HRAS, FBXW7, PIK3AP1, RIMBP2, SI, NRXN2, NRXN3, RASA1, RXFP3*	—
**Stransky et al. (2011)**	38*	White	Tobacco 95%, HPV 5%	—	*TP53, NOTCH1, CDKN2A, FAT1, CASP8, HRAS, PIK3CA, SYNE1, MLL2, CSMD3, SI, PRDM*	—
**Lui et al. (2013)**	24	White	—	—	Only genes in PI3K, MAPK, and JAK/STAT pathways were reported, but specific mutation details were not provided.	—
**Pickering et al. (2013)**	40	White	Tabacco 77%	Tongue (71.4%), floor of the mouth (19%), others (9.5%)	*TP53, FAT1, PIK3CA, CASP8, NOTCH1, HRAS, TP63, NOTCH2, BRAF*	—
**ICGC (2013)**	50	Indian	Tobacco 96%, alcohol 48%, areca nut 36%, HPV 26%	Gingivobuccal 100%	*TP53, FAT1, CASP8, NOTCH1, HRAS, MLL2,* ***USP9X, MLL4, UNC13C, ARID2, TRPM3, PCLO, FAT3, SMG1, SYNE2, EP300***	< 6% representation in TCGA
**TCGA (2015)**	172*	153 White, 11 Black, 2 Asian, 6 unknown	Tobacco 73%, HPV 7%	Tongue 44%, mucosa 28%, floor 15%, gingiva 4%, palate 3%, lip 1%	*TP53, FAT1, CDKN2A, NOTCH1, PIK3CA, CASP8, MLL2 (KMT2D), HRAS, NSD1, FBXW7, AJUBA, HLAA, NFE2L2*	—
**Al-Hebshi et al. (2016)**	20	Arab	Shammah 75%, tabacco 30%, EBV 70%	Tongue 45%, gingiva 20%, floor 15%, lip 10%, others 10%	*CDKN2A, TP53, CASP8, CSMD3, PIK3CA, TP63, FAT1, HRAS,* ***CRB1, NOTCH3, CLTCL1 (15%), OSMR (10%), TRPM2 (10%)***	< 5% representation in TCGA
**Su et al. (2017)**	120	Taiwan	Alcohol 89%, betel nut 79%, tobacco 50%	Oral mucosa 40%, tongue 26.7%, lip 10%, gingiva 9.2%, others 14.1%	*TP53, FAT1, NOTCH1, CASP8, PIK3CA, CDKN2A, HRAS,* ***EPHA2, ELAVL1, CHUK, RPTN, ASXL1***	Lower *TP53*, higher *CASP8* and *FAT1* mutations

**Zammit AP et al. (2018)**	51	White (Australia)	Tobacco 84.3%, alcohol 80.3%, HPV 4.5%	—	*TP53, CDKN2A, SMAD4, PROM1, HP, ALDH1A3, CSK, PARN, C9orf3, DOCK7. NO NOTCH1, KRAS Y PTEN*	No *NOTCH1, KRAS, or PTEN* mutations; transcriptome analysis showed increased mTOR signaling and decreased mitochondrial function in smokers
**Ghias et al. (2019)**	7	Asian (Pakistan)	Betel nut 57.1%, tobacco 14.2%	Oral mucosa 28.5%, gingiva 14.2%, tongue 42.8%, others 14.2%	*TP53, PIK3CA, FGFR2, ARID2, MLL3, MYC, ALK.* ***ASNS***	—
**Campbell et al. (Feb 2021)**	227	USA (Oral Tongue Cancer Consortium) White 65.1%, Indian 22.9%, Asian 11.9%	Tobacco, smokeless tobacco, betel 86.7%	They don't mention subsites. No HPV. Advanced stage 100%	*TP53, CDKN2A, CASP8, NOTCH1, FAT1.* ***ATXN1, CDC42EP1***	Patients under 50 years of age presented low TMB
**Fan WL et al. (30 May 2021)**	165	Taiwan	Smoking (cigarettes and betel) 87%, alcohol 82%	Tongue (71.4%), floor of the mouth (19%), others (9.5%)	*TP53, FAT1, NOTCH1, CASP8, CDKN2A, PIK3CA, HRAS, MUC5B,* ***LINC0027, DNAH5****, MUC4, FAT2,* ***KMT2B, PLEC****, RASA1, SPEN, SYNE1,* ***TGF-β (3.6%)***	*TP53, FAT1, and NOTCH1 were co-mutated in 80.6% of cases. The most frequently altered concurrent signaling pathway was TP53 and RTK/RAS/MAPK*.<br>Overall survival (OS) in mutations: *CDKN2A* had a relative risk (RR) of 2.1 for OS ($p \leq 0.001$).<br>OS in signaling pathways: TP53 and NOTCH ($p = 0.0081$), RTK/RAS/MAPK and NOTCH ($p = 0.0018$), RTK/RAS/MAPK and cell cycle ($p = 0.0008$).<br>Disease-free survival (DFS) in signaling pathways: Hippo and NOTCH ($p = 0.0075$), NOTCH and PI3K ($p = 0.0076$), NOTCH and WNT ($p = 0.0025$), Cell cycle and PI3K ($p = 0.0051$).<br>OS and DFS<br>NOTCH, RTK/RAS/MAPK, and TGF-beta were associated with OS ($p = 0.0120$) and PFS ($p = 0.0009$).<br>30% of tumors presented actionable therapeutic targets
**Patel el tal. (31 Mayo 2021)**	30	India	Tobacco 33%, chewing 33%	Oral mucosa 36.7%, tongue 26.7%, lip 10%, gingiva 10%, others 16.7%. 70% Stage III-IV	*TP53, NOTCH1, CDKN2A, FAT1, PIK3CA y HRAS*	*NOTCH1 36%* and *HRAS 13%*<br>Signature SBS 29 detected in 40% of OSCC samples from tobacco chewers. Signature SBS 15 (mismatch repair) found in 80%.
**Liao CT et al. (Oct 2021)**	168	Taiwan	Tobacco 86.3%, alcohol 81.5%, betel 86.3%	They don't mention subsites. Only surgically treated patients Stage IV 87.5%	*TP53, FAT1, NOTCH1 ,* ***TTN****, CASP8 , CDKN2A, HRAS,* ***ZFHX4, DNAH5****, KMT2B, PIK3CA, RASA1 y FAT2*	Nine-gene panel (*RYR1, HLA-B, TSHZ2, PCDH17, DNAH17, GRID1, SBNO2, KSR2, GCN1L1*), impact on prognosis.<br>Signature SBS 13, 1, and 7.

**Lin LH et al. (Nov 2021)**	50	Taiwan	—	Oral mucosa 28%, gingiva 24%<br>N+ 44%<br>Stage IV 78%	*TP53, FAT1 , EPHA2 , NOTCH1 , CASP8 y PIK3CA.* ***HYDIN , ALPK3 , ASXL1 , USP9X , SKOR2 , CPLANE1 , STARD9 y NSD2.***	*CASP8, USP9X,* and *FAT1* associated with worse overall survival (OS) (p=0.004, p=0.018, p=0.050).<br>High tumor mutational burden (TMB) correlated with advanced clinical stage and lower survival (p=0.041).<br>Involvement of NF-Kappa-B pathways, calcium signaling, and clathrin-mediated endocytosis.<br>54% of cases are potentially treatable with targeted therapies

* Studies included other anatomical head and neck locations.
HPV: Human papillomavirus
EBV: Epstein-Barr virus
ICGC: International Cancer Genome Consortium
TCGA: The Cancer Genome Atlas
The genes highlighted in bold represent unique genes in those populations
