# Supplementary material for: Molecular Characterization of Oral Squamous Cell Carcinoma in Mexican Patients: A Genomic and Epidemiological Overview
Source: Cancers (Basel). 2025 Oct 10;17(20):3282. doi: 10.3390/cancers17203282 (PMC12564337; doi:10.3390/cancers17203282)
Supplement: Supplementary file 1 [file cancers-17-03282-s001.zip › Table S2.pdf]

Table S2. Main Functions of Proteins in Pathogenic Variants Identified in OSCC Sequencing Analysis					
**Gene**	**%**	**Affected Patients**	**Function**	**Comparison with Previous Studies (Table 1)**	**Comparison with TCGA (%)**
TP53	51	02, 03, 04, 06, 10, 11, 16, 17, 18, 19, 20, 23, 25, 26, 28, 30, 31, 32, 35, 37, 42, 43, 47, 49, 52, 53, 54 y 55	The encoded protein responds to various cellular stimuli to regulate gene expression, inducing cell cycle arrest, apoptosis, senescence, DNA repair, or metabolic changes.	Described in all studies	74.2
FAT1	20	01, 04, 05, 11, 13, 20, 30, 36, 43, 46 y 52	The gene product belongs to the cadherin superfamily, a group of transmembrane proteins involved in cell adhesion. It is highly expressed in various fetal epithelia and likely functions as a tumor suppressor.	Described in the following studies: Stransky et al., ICGC, TCGA, Al-Hebshi et al., Su et al., Campbell et al., Fan WL et al., Patel et al., Liao CT et al., Lin LH et al.	27.6
KMT2C	18	01, 15, 21, 22, 32, 39, 40, 47, 49 y 54	This gene belongs to the Mixed-Lineage Leukemia (MLL) family and encodes a nuclear protein, part of the ASC-2/NCOA6 (ASCOM) complex, with histone methyltransferase activity involved in transcriptional coactivation.	Not described in previous studies	5.1
NOTCH1	18	04, 05, 14, 16, 17, 28, 36, 39, 46, 54,	Encodes a type I transmembrane receptor involved in the Notch signaling pathway, regulating interactions between adjacent cells by binding to Notch family ligands. Plays a crucial role in cell differentiation and tumor progression	Described in the following studies: Agrawal et al.<br>Stransky et al.<br>Pickering et al.<br>ICGC<br>TCGA<br>Su et al.<br>Campbell et al.<br>Fan WL et al.<br>Patel et al.<br>Liao CT et al.<br>Lin LH et al.	20.9
CDKN2A	15	05, 28, 36, 37, 47, 49, 52 y 55	Encodes a tumor suppressor protein that stabilizes p53 by interacting with and sequestering the E3 ubiquitin-protein ligase MDM2, which regulates p53 degradation, controlling the G1 phase of the cell cycle.	Described in the following studies: Agrawal et al.<br>Stransky et al.<br>TCGA<br>Al-hebshi et al.<br>Su et al.<br>Zammit AP et al.<br>Campbell et al.<br>Fan WL et al.<br>Patel et al.<br>Liao CT et al.	23.4
CASP8	13	03, 36, 40, 43, 46, 48 y 54	Encodes Caspase-8, a crucial enzyme in the apoptotic pathway activated via the Fas receptor.	Described in the following studies: Stransky et al.<br>Pickering et al.<br>ICGC<br>TCGA<br>Al-hebshi et al.	15.7

				Su et al.<br>Campbell et al.<br>Fan WL et al.<br>Liao CT et al.<br>Lin LH et al.	
MUC16	13	21, 31, 32, 38, 44, 48 y 52	Encodes a mucin family protein involved in forming a protective mucosal barrier on epithelial surfaces, preventing pathogen invasion.	Not described in previous studies	17.3
NOTCH2	9	32, 44, 47, 49 y 52	Similar to NOTCH1, encodes a type I transmembrane receptor involved in the Notch pathway, regulating intercellular signaling and tumor development.	Described in the following studies: Pickering et al., Liao CT et al.	5.1
ARID1A	7	12, 30, 41 y 52	Encodes a chromatin remodeling complex SWI/SNF component, regulating gene expression by altering chromatin structure. Plays a role in transcriptional regulation, cell cycle control, tumor suppression, and immune response modulation.	Not described in previous studies	3.2
ARID2	7	15, 21, 31 y 46	Encodes a DNA-binding protein that functions in embryonic patterning, cell lineage regulation, cell cycle control, transcriptional regulation, and chromatin remodeling. Mutations are associated with hepatocellular carcinoma.	Described in the following studies: ICGC<br>Ghias et al.	3.5
BIRC6	7	01, 18, 47 y 52	Encodes Baculoviral IAP repeat-containing protein 6 (BIRC6), which inhibits apoptosis by interfering with caspases and functions as an E3 ubiquitin ligase, regulating protein degradation via the proteasome.	Not described in previous studies	5.1
DCC	7	04, 27, 48 y 49	Encodes a netrin-1 receptor, playing a crucial role in neuronal growth, apoptosis regulation, tumor suppression, and cell migration/adhesion.	Not described in previous studies	4.1
NCOR1	7	14, 29, 39 y 50	Encodes a nuclear receptor corepressor, forming complexes with histone deacetylases (HDACs) to modify chromatin and repress gene transcription. Plays a role in adipocyte differentiation, neural regulation, immune cell differentiation, and metabolic homeostasis.	Not described in previous studies	3.2
