# Supplementary material for: Molecular Characterization of Oral Squamous Cell Carcinoma in Mexican Patients: A Genomic and Epidemiological Overview
Source: Cancers (Basel). 2025 Oct 10;17(20):3282. doi: 10.3390/cancers17203282 (PMC12564337; doi:10.3390/cancers17203282)
Supplement: Supplementary file 1 [file cancers-17-03282-s001.zip › Table S3.pdf]

Table S3. Single Base Substitution (SBS) Signatures in Oral Squamous Cell Carcinoma (OSCC)			
**SBS Signature**	**Cases (%)**	**Mutational Processes Involved**	**Clinical and Molecular Associations**<br><br>**(Student's T-test, Mann-Whitney U, Chi-square)**
SBS 5	24 (43.6)	Unknown. Proposed: Aging, smoking, and nucleotide excision repair deficiency. Associated with bladder cancer.	Presence of periodontal disease (p = 0.004).<br>Higher recurrence risk (p = 0.016).<br>Lower TMB (p = 0.035).<br>Lower prevalence of *MUC16* mutation (p = 0.015)
SBS 1	12 (21.8)	Deamination of 5-methylcytosine (Aging). Associated with SBS 5.	No significant association with age or smoking.<br>Negative nodal status (p=0.020).
SBS 13	9 (16.4)	APOBEC-related signature. Viral infection and inflammation. Associated with breast and bladder cancer. Related to SBS 2.	-
SBS 12	8 (14.5)	Unknown. Found in <20% of hepatocellular carcinoma cases.	Presence of *NIN* mutation (p=0.019) (only two cases documented).
SBS 37	8 (14.5)	Unknown	Absence of periodontal disease (p=0.002).
SBS 3	6 (10.9)	Defective DNA repair (linked to BRCA1/2). Associated with breast, ovarian, and pancreatic cancer.	Greater number of TMB (p=0.033)<br>Periodontal disease (p=0.048).<br>Lower prevalence of *NOTCH2* mutation (p=0.007), *ABCB4* and *MGA* mutations (p=0.029).
SBS 7a	6 (10.9)	Ultraviolet light exposure. Associated with melanoma. Linked to ID-13.	Lower prevalence of *CASP8* mutation (p=0.022).
SBS 40a	6 (10.9)	Unknown. Associated with clear cell renal carcinoma.	Negative nodal status (p=0.020).<br>Absence of *CYTH4* mutation (p=0.029).
SBS 15	5 (9.1)	Defective DNA repair and microsatellite instability (MSI). Associated with ID-01 and ID-02.	High TMB (p=0.033).<br>*KMT2C* mutation (p=0.037).
SBS 22a	5 (9.1)	Aristolochic acid exposure. Associated with hepatocellular, renal, biliary, esophageal, and bladder carcinomas.	-
SBS 96	5 (9.1)	Unknown. Rarely detected in renal carcinoma.	Presence of periodontal disease (p = 0.047).<br>Presence of sarcomatoid component (p=0.012).
**Tobacco-Related Signature**	28 (50.9)	SBS 4 y SBS 5 (likely)	No significant association with smoking.<br>Presence of periodontal disease (p=0.009).<br>Lymphovascular invasion (p=0.026).<br>Increased recurrence risk (p=0.029).<br>Lower TMB (p=0.019).<br>Lower prevalence of *MUC16* mutation (p=0.045)
**MSI Signature**	11 (20)	SBS 3, 6, 15 y 26	Presence of periodontal disease (p=0.005).<br>Lower prevalence of *NOTCH2* mutation (p=0.049).
**APOBEC Signature**	9 (16.3)	SBS 2 y 13	-
**Ultraviolet Radiation Signature**	8 (14.5)	SBS 7a, b, c, d y 38	Presence of periodontal disease (p=0.037).
SBS 2	4 (7.3)	APOBEC-mediated mutation. Previous viral infection and inflammation. Related to SBS 13.	-
SBS 4	4 (7.3)	Smoking-related exposure to benzo[a]pyrene. Direct DNA damage. SBS	-

		29 found in tobacco-associated cancers. Associated with ID-03.	
SBS 18	4 (7.3)	Reactive oxygen species (ROS)-induced damage.	-
SBS 89	4 (7.3)	Unknown. Found in early life (first decade). Identified in normal colorectal epithelial cells.	-
SBS 26	3 (5.5)	Defective DNA repair and microsatellite instability (MSI). Associated with ID-1 and ID-2.	-
SBS 42	3 (5.5)	Occupational exposure to haloalkanes (semiconductor manufacturing, refrigerants, foaming agents, solvents, aerosol propellants, extinguishing agents, and chemical reagents).	-
SBS 54	3 (5.5)	Possible sequencing artifact. Potential contamination with germline variants.	-
SBS 94	3 (5.5)	Unknown. Identified in colorectal cancer samples.	-
SBS 6	3 (5.5)	Defective DNA repair and MSI. Associated with ID-1 and ID-2.	-
SBS 29	2 (3.6)	Chewing tobacco exposure.	-
SBS 7b	2 (3.6)	Ultraviolet light exposure. Associated with melanoma. Related to ID-13.	-
SBS 7d	2 (3.6)	Ultraviolet light exposure.	-
SBS 23	2 (3.6)	Unknown.	-
SBS 58	2 (3.6)	Possible sequencing artifact.	-
SBS 92	2 (3.6)	Tobacco-related signature. Associated with bladder cancer, SBS 4, and ID-3.	-
SBS 97	2 (3.6)	Unknown. Similar to SBS 7b, associated with skin and connective tissue cancers.	-
SBS 30	1 (1.8)	Base excision repair deficiency due to inactivating mutations in NTHL1.	-
SBS 7c	1 (1.8)	Ultraviolet light exposure. Associated with melanoma. Related to ID-13.	-
SBS 25	1 (1.8)	Unknown. Likely related to chemotherapy exposure (Hodgkin's lymphoma).	-
SBS 8	1 (1.8)	Unknown.	-
SBS 33	1 (1.8)	Unknown.	-
SBS 38	1 (1.8)	Indirect UV damage. Found exclusively in melanoma, along with SBS 7a and 7b.	-
SBS 39	1 (1.8)	Unknown.	-
SBS 40c	1 (1.8)	Unknown. Identified in clear cell renal carcinoma.	-
SBS 45	1 (1.8)	Possible artifact due to 8-oxoguanine introduction during sequencing.	-
SBS 57	1 (1.8)	Possible sequencing artifact.	-
SBS 84	1 (1.8)	Cytidine deaminase activity induced by activation. Associated with immunoglobulin genes and other regions in lymphoid malignancies.	-

SBS 87	1 (1.8)	Thiopurine chemotherapy exposure. Identified in a relapsed acute lymphoblastic leukemia (ALL) patient.	-
SBS 90	1 (1.8)	Duocarmycin exposure. Found in metastatic breast and esophageal cancers.	-
SBS 95	1 (1.8)	Possible sequencing artifact.	-

Green: Validated signature with strong evidence

Yellow: Uncertain evidence of a true mutational signature

Red: Potential sequencing artifact

NHEJ: Non-Homologous End Joining (NHEJ) DNA Repair Mechanism

n: number of patients

OR: Odds Ratio

TMB: Tumor Mutational Burden

https://cancer.sanger.ac.uk/signatures/ (12)
