# Supplementary material for: Molecular Characterization of Oral Squamous Cell Carcinoma in Mexican Patients: A Genomic and Epidemiological Overview
Source: Cancers (Basel). 2025 Oct 10;17(20):3282. doi: 10.3390/cancers17203282 (PMC12564337; doi:10.3390/cancers17203282)
Supplement: Supplementary file 1 [file cancers-17-03282-s001.zip › Table S4.pdf]

Table S4. Insertion and Deletion (InDel) Signatures

InDel Signature	Cases (%)	Mutational Processes Involved	Clinical and Molecular Associations<br><br>(Student's t-test, Mann-Whitney U test, and Chi-square test)
ID-19	19<br>(34.5%)	Unknown (5 base pair insertions)	Non-exposure to tobacco and alcohol (p = 0.011).<br>Periodontal disease (p = 0.001).<br>Higher prevalence of *KMT2C, MUC16, and NOTCH2* (p = 0.023, <0.001, and 0.003).<br>Higher TMB (p < 0.001).<br>Lower prevalence of tobacco-related signatures (SBS4 and SBS5): (p = 0.037).<br>Lower prevalence of SBS 5 (p = 0.002).
ID-08	8<br>(14.5)	DNA double-strand break repair via NHEJ or TOP2A mutations. Associated with renal clear cell carcinoma in older patients. Correlated with SBS 40b and ID-05 signatures.	Absence of tobacco-related signatures (SBS4 and SBS5): (p = 0.002).<br>MSI signatures (p = 0.042).<br>UV-related signatures (p = 0.011).
ID-12	7<br>(12.7)	Unknown	Periodontal disease (p = 0.011).
ID-10	6<br>(10.9)	Unknown	Absence of Periodontal disease (p = 0.002).<br>Lower number of mutations/Mb, (p = <0.001).<br>Higher prevalence of *RASA1* (p = 0.029).
ID-06	6<br>(10.9)	Homologous DNA repair defect associated with BRCA1/BRCA2. Related to SBS 3.	Presence of SBS 1 (p = 0.017).<br>Presence of SBS 42 (p = 0.029).
ID-01	5<br>(9.1)	DNA strand slippage during replication. Age-related. Microsatellite instability (MSI). Associated with SBS 6, SBS 14, SBS 20, SBS 21, SBS 26, and SBS 44.	Early clinical stage disease (p = 0.027).
ID-02	5<br>(9.1)	DNA strand slippage during replication. Age-related. MSI. Associated with SBS 6, SBS 14, SBS 20, SBS 21, SBS 26, and SBS 44.	Absence of periodontal disease (p = 0.005).<br>Lower number of mutations/Mb (p =< 0.001).<br>Higher prevalence of *RASA1* (p = 0.019).<br>Higher prevalence of tobacco-related signatures (SBS4 and SBS5): (p = 0.028).
ID-04	5<br>(9.1)	Unknown	Lower number of mutations/Mb (p =< 0.001).
ID-03	2<br>(3.6)	Tobacco exposure. Associated with SBS 4 and DBS 2.	-
ID-07	2<br>(3.6%)	Mismatch repair deficiency (MSI). Associated with ID-01 and ID-02.	-
ID-09	2<br>(3.6%)	Unknown	-
ID-21	2<br>(3.6%)	Unique signature composed of 2-4 bp deletions in double repeats. Common in the central nervous system.	-
ID-23	2<br>(3.6%)	Aristolochic acid exposure. Associated with renal clear cell carcinoma. Related to SBS 22a, SBS 22b, and DBS 20.	-
ID-05	1<br>(1.8%)	Unknown. Age-related. Associated with SBS40b and ID-08. Linked to renal clear cell carcinoma.	-
ID-14	1<br>(1.8%)	Unknown	-

ID-17	1 (1.8%)	TOP2A topoisomerase mutations. Associated with ID-08.	-

Green: Validated signature with strong evidence

Yellow: Uncertain evidence of a true mutational signature

Red: Potential sequencing artifact

NHEJ: Non-Homologous End Joining (NHEJ) DNA Repair Mechanism

TMB: Tumor Mutational Burden

https://cancer.sanger.ac.uk/signatures/ (12)
