# Supplementary figures and images for: Molecular Characterization of Oral Squamous Cell Carcinoma in Mexican Patients: A Genomic and Epidemiological Overview
Source: Cancers (Basel). 2025 Oct 10;17(20):3282. doi: 10.3390/cancers17203282 (PMC12564337; doi:10.3390/cancers17203282)

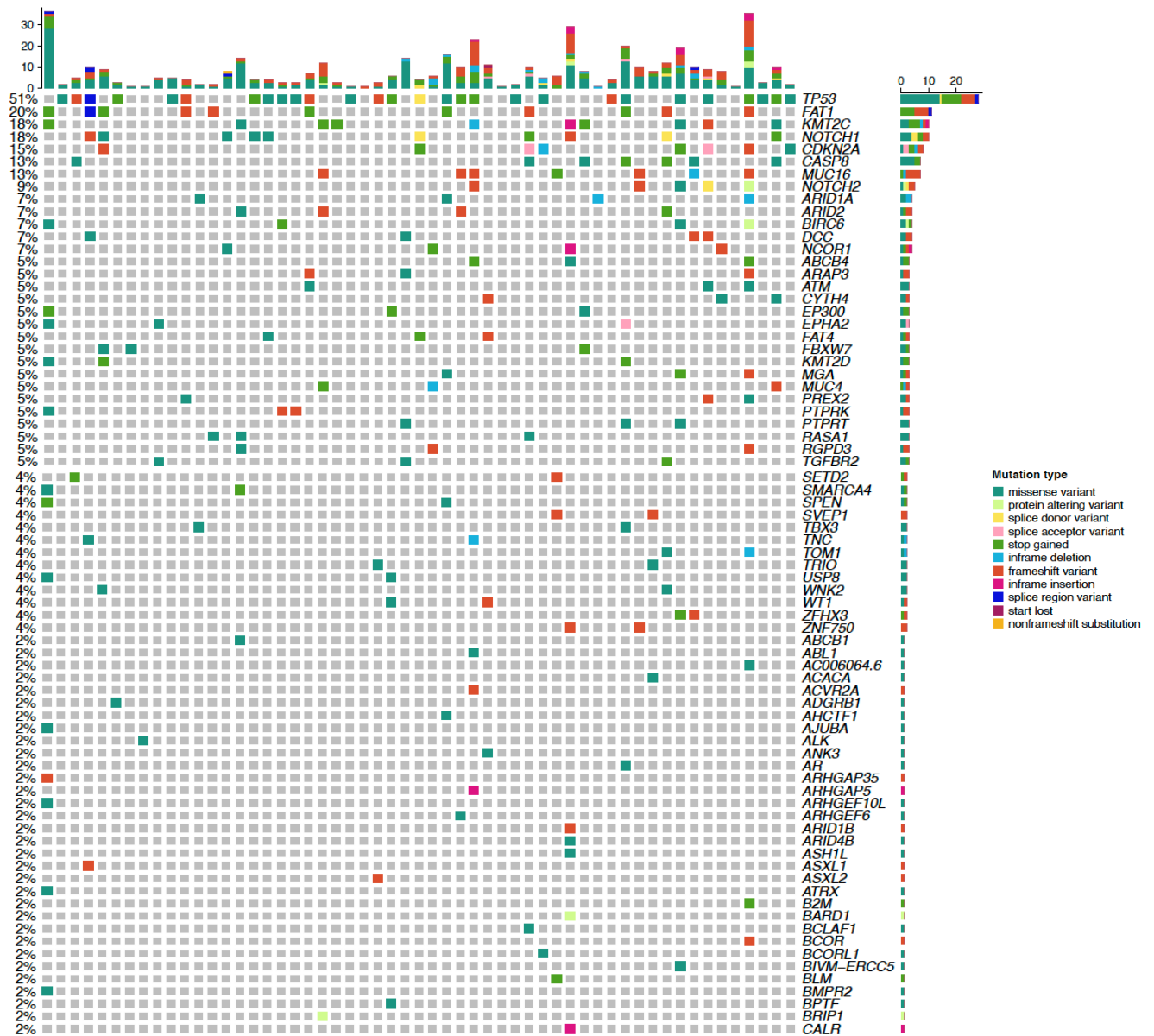



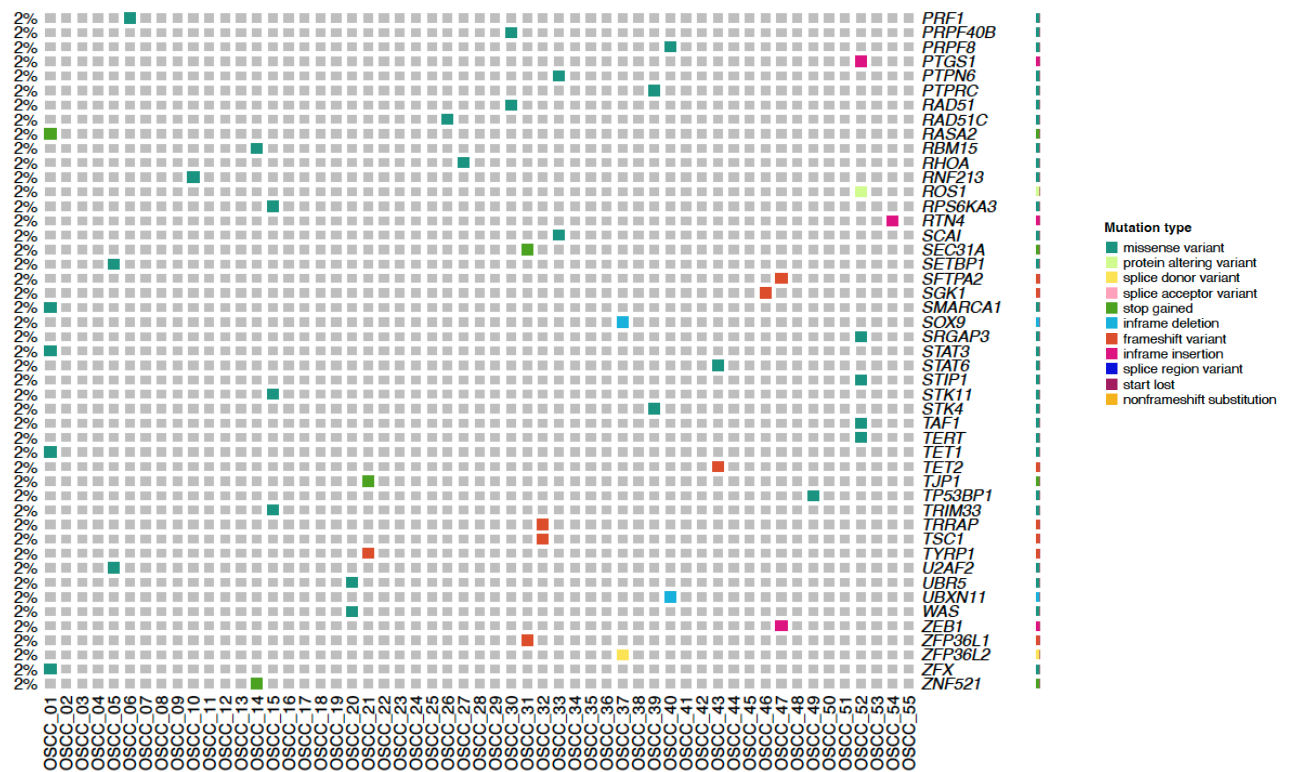

Supplement: Supplementary file 1 [file cancers-17-03282-s001.zip › Figure S1.pdf]
